# Body Composition Changes With Standard Versus Low-Dose Tirzepatide Titration in Women With Lipedema: A Retrospective Observational Study

**DOI:** 10.7759/cureus.116779

**Published:** 2026-09-23

**Authors:** Paula Frederichi de Souza, Gustavo Patriani, Camila Maria Ribeiro Pacheco

**Affiliations:** 1 Scientific Department, Educational Organization in Lipedema (ABRALIPRO), São Paulo, BRA; 2 Nutrition Department, Instituto Paula Frederich, São Paulo, BRA

**Keywords:** anthropometry, bioelectrical impedance analysis, fat distribution, fat mass, incretin therapy, lean mass

## Abstract

Purpose: This study aimed to compare 12-week changes in body composition and anthropometric parameters between standard-dose and lower-dose tirzepatide escalation regimens in women with lipedema, with particular emphasis on changes in fat mass and skeletal muscle mass.

Patients and methods: This retrospective observational study included 100 women with lipedema receiving tirzepatide during routine clinical care. Participants were classified into lower-dose (n = 50) and standard-dose (n = 50) groups according to their prescribed dosing regimens, which differed in cumulative drug exposure. Body composition, anthropometric measurements, and skinfold thickness were assessed at baseline and after 12 weeks. An exploratory composite responder criterion was defined as a ≥5% reduction in fat mass combined with a <5% reduction in skeletal muscle mass, as estimated by bioelectrical impedance analysis (BIA). Between-group comparisons were unadjusted.

Results: Both regimens were associated with reductions in body weight, fat mass, and body fat percentage (all within-group p < 0.001). No statistically significant between-group differences were detected in these changes. The lower-dose regimen was associated with a smaller reduction in BIA-estimated skeletal muscle mass than the standard-dose regimen (−0.7 vs. −2.0 kg; p < 0.001). The exploratory composite responder criterion was met by 68% (34/50) of participants in the lower-dose group and 22% (11/50) in the standard-dose group (OR = 7.53; 95% CI: 3.08-18.44; p < 0.001). No statistically significant between-group differences were detected in anthropometric measurements or skinfold thickness changes.

Conclusion: Both tirzepatide dosing regimens were associated with reductions in body weight and adiposity in women with lipedema. The lower-dose regimen was associated with a smaller reduction in BIA-estimated skeletal muscle mass and a higher proportion of participants meeting the exploratory composite responder criterion. These findings require confirmation in prospective controlled studies, given the non-randomized design, differences in cumulative drug exposure, and potential residual confounding.

## Introduction

Body weight alone may provide an incomplete representation of treatment-related changes in women with lipedema. Body mass index (BMI) does not distinguish fat mass from lean mass and may not adequately characterize the disproportionate distribution of adipose tissue typical of the condition [1-3]. Metabolic characteristics may also differ from those expected on the basis of BMI alone. Compared with women with lifestyle-induced overweight or obesity, women with lipedema showed a lower prevalence of impaired glucose metabolism and insulin resistance despite elevated BMI [4]. Taken together, these characteristics indicate that body weight and BMI alone may not adequately represent metabolic status or changes in body composition in this population.

The distinction between weight loss and changes in body composition becomes particularly important when weight reduction is part of clinical management. Women with lipedema may present with impairments in lower-limb muscle strength and endurance, functional performance, and exercise capacity [5]. Substantial reductions in body weight do not necessarily resolve characteristic symptoms, as persistent pain has been reported after marked weight loss following bariatric surgery [6]. Dietary weight-loss interventions in women with lipedema and obesity have also resulted in reductions in fat-free mass and muscle area alongside reductions in body weight and fat mass [7]. These observations highlight the importance of evaluating changes in individual body-composition components during weight-loss interventions rather than relying exclusively on total body weight. Current clinical recommendations similarly incorporate healthy body composition, mobility, and functional capacity into weight-management goals for women with lipedema [8].

Tirzepatide, a dual glucose-dependent insulinotropic polypeptide (GIP) and glucagon-like peptide-1 (GLP-1) receptor agonist, promotes marked reductions in body weight and adiposity [9]. In a dual-energy X-ray absorptiometry (DXA) substudy of the SURMOUNT-1 randomized clinical trial, 72 weeks of tirzepatide treatment resulted in mean reductions of 21.3% in body weight, 33.9% in fat mass, and 10.9% in lean mass; approximately 75% of the weight lost was attributable to fat mass and 25% to lean mass [10]. Available randomized evidence also supports reductions in total and visceral adiposity during tirzepatide treatment [11]. However, DXA-derived lean mass and skeletal muscle mass estimated by bioelectrical impedance analysis (BIA) represent distinct body-composition parameters and should not be interpreted interchangeably. These considerations highlight the importance of clearly defining body-composition outcomes when investigating changes associated with tirzepatide treatment.

Dose escalation is an integral component of tirzepatide treatment, with therapy initiated at a low dose and progressively increased, in part to improve gastrointestinal tolerability [9]. Different dose-escalation regimens have been associated with distinct gastrointestinal tolerability profiles in individuals with type 2 diabetes [12]. However, evidence regarding body-composition changes associated with different tirzepatide dosing regimens remains limited, particularly in women with lipedema. Importantly, regimens involving different dose-escalation schedules may also result in different cumulative drug exposures, making it difficult to distinguish the association of the escalation pattern from that of overall dose exposure.

Therefore, this retrospective observational study aimed to compare 12-week changes in body composition and anthropometric parameters between women with lipedema receiving standard-dose and lower-dose tirzepatide escalation regimens, with particular emphasis on changes in fat mass and BIA-estimated skeletal muscle mass. The comparison involved regimens that differed in both dose escalation and cumulative drug exposure.

## Materials and methods

Study design and participants

This retrospective comparative observational study was conducted at a specialized clinical center for women with lipedema in São Paulo, Brazil, using medical records from patients treated between June 2024 and June 2025. A total of 100 women who completed 12 weeks of clinical follow-up and met the eligibility criteria were included in the analysis.

Lipedema was diagnosed clinically based on medical history and physical examination, considering characteristic features such as disproportionate, bilateral, and predominantly symmetrical adipose tissue accumulation in the extremities, relative sparing of the feet, and associated pain or tenderness.

Eligible participants were women with a clinical diagnosis of lipedema who had anthropometric and body-composition assessments available at baseline and after 12 weeks of follow-up. Patients with previous use of incretin-based therapies or insulin, a history of pancreatitis, uncontrolled hypothyroidism, bariatric surgery within the preceding 12 months, severe gastrointestinal disease, pregnancy, or lactation were excluded.

The study was approved by the Research Ethics Committee of Universidade Presbiteriana Mackenzie, São Paulo, Brazil (Approval No. 8.052.687; CAAE: 94490425.4.0000.0084). As the study was based exclusively on secondary data obtained from medical records, no direct patient contact occurred, and no additional interventions were performed for research purposes.

Tirzepatide treatment and titration strategies

Participants were retrospectively classified into two groups according to the tirzepatide dosing regimen documented in their medical records during routine clinical follow-up. Treatment regimens were physician-directed and individualized based on clinical judgment and patient tolerability rather than assigned for research purposes.

Compounded tirzepatide was used to allow individualized dosing, particularly the 1.25 mg and 3.75 mg doses employed in the lower-dose regimen. All formulations were obtained from the same licensed compounding pharmacy.

Fifty participants followed a standard-dose escalation regimen consisting of 2.5 mg once weekly during weeks 1-4, 5.0 mg once weekly during weeks 5-8, and 7.5 mg once weekly during weeks 9-12. The remaining 50 participants followed a lower-dose escalation regimen consisting of 1.25 mg once weekly during weeks 1-4, 2.5 mg once weekly during weeks 5-8, and 3.75 mg once weekly during weeks 9-12 (Figure 1). 

**Figure 1:**
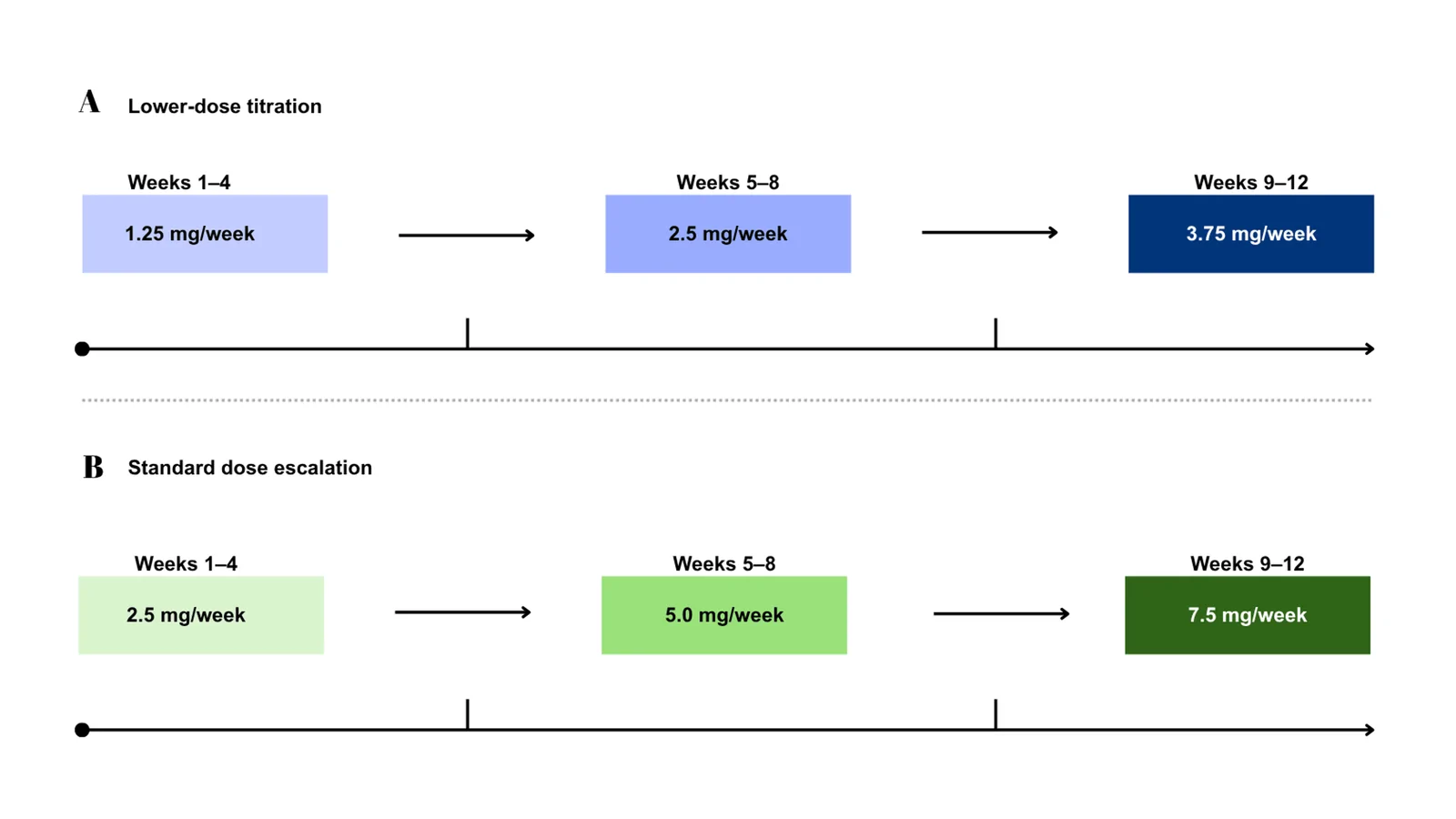
Tirzepatide dosing regimens over the 12-week follow-up period. The figure illustrates the lower-dose titration regimen (A) and the standard dose-escalation regimen (B).

The regimens corresponded to cumulative prescribed doses of 60 mg and 30 mg over 12 weeks for the standard-dose and lower-dose groups, respectively, assuming full adherence to the scheduled weekly administrations. Thus, the groups differed in both dose escalation and cumulative prescribed drug exposure.

Prescribed doses and titration schedules were obtained from medical records. Information regarding treatment adherence and dose modifications was reviewed when documented during routine clinical follow-up.

Throughout the 12-week follow-up period, all participants received standardized qualitative anti-inflammatory dietary counseling under multidisciplinary supervision.

Demographic and clinical information, including age, body weight, body mass index (BMI), anthropometric measurements, body-composition parameters, and clinical follow-up data, was extracted from medical records.

Outcome measures

Body composition was assessed at baseline and after 12 weeks using multifrequency bioelectrical impedance analysis (InBody 120, InBody Co., Seoul, South Korea). The primary outcomes were changes in body fat mass (kg), body fat percentage (%), and BIA-estimated skeletal muscle mass (kg).

A standardized measurement protocol was followed at both assessments. Participants underwent a minimum fasting period of four hours before each examination. Hydration instructions were provided in advance, and compliance was checked before the measurements. Both assessments were performed using the same device and were conducted by the same trained operator.

Skeletal muscle mass was defined as the corresponding device-derived estimate, expressed in kilograms, and was distinguished from total fat-free mass.

Secondary outcomes included changes in body weight (kg), body mass index (BMI, kg/m²), waist, thigh, and arm circumferences, and skinfold thickness at the thigh, abdomen, and triceps sites.

Waist circumference was measured at the midpoint between the lowest rib and the iliac crest; thigh circumference at the midpoint between the inguinal fold and the proximal border of the patella; and arm circumference at the midpoint between the acromion and the olecranon process, with the upper limb relaxed.

An exploratory composite responder criterion was defined as a reduction of at least 5% in body fat mass accompanied by a reduction of less than 5% in BIA-estimated skeletal muscle mass from baseline to 12 weeks. Percentage changes were calculated relative to baseline values. Participants meeting both criteria were classified as responders. These thresholds were established as pragmatic operational criteria for this study and have not been clinically validated as indicators of favorable body-composition response in women with lipedema.

Statistical analysis

Data were reviewed for consistency and completeness before statistical analysis. Exact duplicate records were removed, and implausible values identified during data verification were treated as missing. Analyses were conducted using available paired observations for each outcome, without imputation of missing data.

Continuous variables were summarized as mean ± standard deviation (SD), and categorical variables as frequencies and percentages. Data distribution was assessed using the Shapiro-Wilk test. Given the distributional characteristics of the data, nonparametric methods were used for inferential comparisons.

Within-group changes from baseline to 12 weeks were assessed using the Wilcoxon signed-rank test. Changes were calculated as the 12-week value minus the baseline value. Between-group comparisons of baseline characteristics and changes from baseline were performed using the Mann-Whitney U test.

The exploratory composite responder analysis was conducted using the study-defined criteria described in the Outcome Measures section. Responder proportions were compared between dosing regimens using Fisher's exact test. The association between dosing regimen and responder status was expressed as an odds ratio (OR) with a 95% confidence interval (CI) [13].

All analyses were unadjusted, and no multivariable adjustment was performed. Information on several potentially relevant confounding variables, including physical activity, dietary intake, protein consumption, and lipedema severity, was not systematically available in the retrospective medical records.

All statistical tests were two-sided, and statistical significance was set at p < 0.05. Statistical analyses were performed using Minitab Statistical Software (Minitab LLC, State College, Pennsylvania).

## Results

Baseline characteristics of the study population

Baseline demographic, anthropometric, and body-composition characteristics showed no statistically significant between-group differences in age, body weight, body mass index (BMI), fat mass, body fat percentage, or BIA-estimated skeletal muscle mass (all p > 0.05). However, standardized mean differences indicated some baseline imbalance in age despite the absence of statistical significance (Table 1).

**Table 1:** Baseline demographic and body composition characteristics according to tirzepatide titration strategy. Data are presented as mean ± standard deviation (SD). Between-group comparisons were performed using the Mann–Whitney U test. Absolute standardized mean differences (SMDs) were calculated using the pooled standard deviation. Skeletal muscle mass was estimated using bioelectrical impedance analysis (BIA). Statistical significance was set at p < 0.05.

Characteristic	Lower dose (n = 50)	Standard dose (n = 50)	p-value	Absolute SMD
Age (years)	44.56 ± 10.08	40.74 ± 12.23	0.128	0.34
Body weight (kg)	84.85 ± 14.87	83.49 ± 13.48	0.714	0.10
BMI (kg/m²)	31.70 ± 5.40	31.14 ± 4.72	0.933	0.11
Fat mass (kg)	34.52 ± 10.28	34.96 ± 10.42	0.889	0.04
Body fat (%)	40.33 ± 7.95	41.56 ± 7.34	0.482	0.16
Skeletal muscle mass (kg)	27.84 ± 6.16	27.76 ± 5.71	0.973	0.01

Changes in body weight and adiposity

After 12 weeks, statistically significant reductions in body weight, fat mass, and body fat percentage were observed within both dosing groups compared with baseline (all p < 0.001). No statistically significant between-group differences were detected in changes in body weight (p = 0.084), fat mass (p = 0.250), or body fat percentage (p = 0.558). These findings indicate that both regimens were associated with reductions in body weight and adiposity, without establishing equivalence between the groups (Table 2).

**Table 2:** Changes in body weight and adiposity from baseline to 12 weeks according to tirzepatide titration strategy. Data are presented as mean change ± standard deviation (SD). Negative values indicate reductions from baseline to 12 weeks. Statistical significance was set at p < 0.05.

Outcome	Lower-dose Δ mean ± SD	Standard-dose Δ mean ± SD	Mean difference (95% CI)	p-value
Body weight (kg)	−8.20 ± 5.14	−9.73 ± 4.99	1.53 (−0.48 to 3.54)	0.084
Fat mass (kg)	−6.46 ± 5.32	−6.98 ± 4.89	0.52 (−1.51 to 2.55)	0.250
Body fat (percentage points)	−4.48 ± 5.32	−4.79 ± 4.68	0.31 (−1.68 to 2.30)	0.558

Muscle mass preservation and responder analysis

Despite comparable reductions in body weight and adiposity, changes in muscle mass differed significantly between titration regimens. Muscle mass decreased significantly in both groups over the 12-week follow-up; however, the reduction was smaller in the lower-dose titration group than in the standard dose-escalation group (−0.7 kg vs. −2.0 kg, respectively; p < 0.001 for the between-group comparison).

Consistent with this finding, the defined favorable body composition response was more frequent in the lower-dose titration group. Among participants with complete fat mass and muscle mass data, 68% (34/50) of those receiving the lower-dose titration regimen met the responder criterion, compared with 22% (11/50) in the standard dose-escalation group. The lower-dose titration regimen was associated with significantly greater odds of achieving the defined favorable body composition response (OR = 7.53; 95% CI: 3.08-18.44; p < 0.001) (Figure 2).

**Figure 2:**
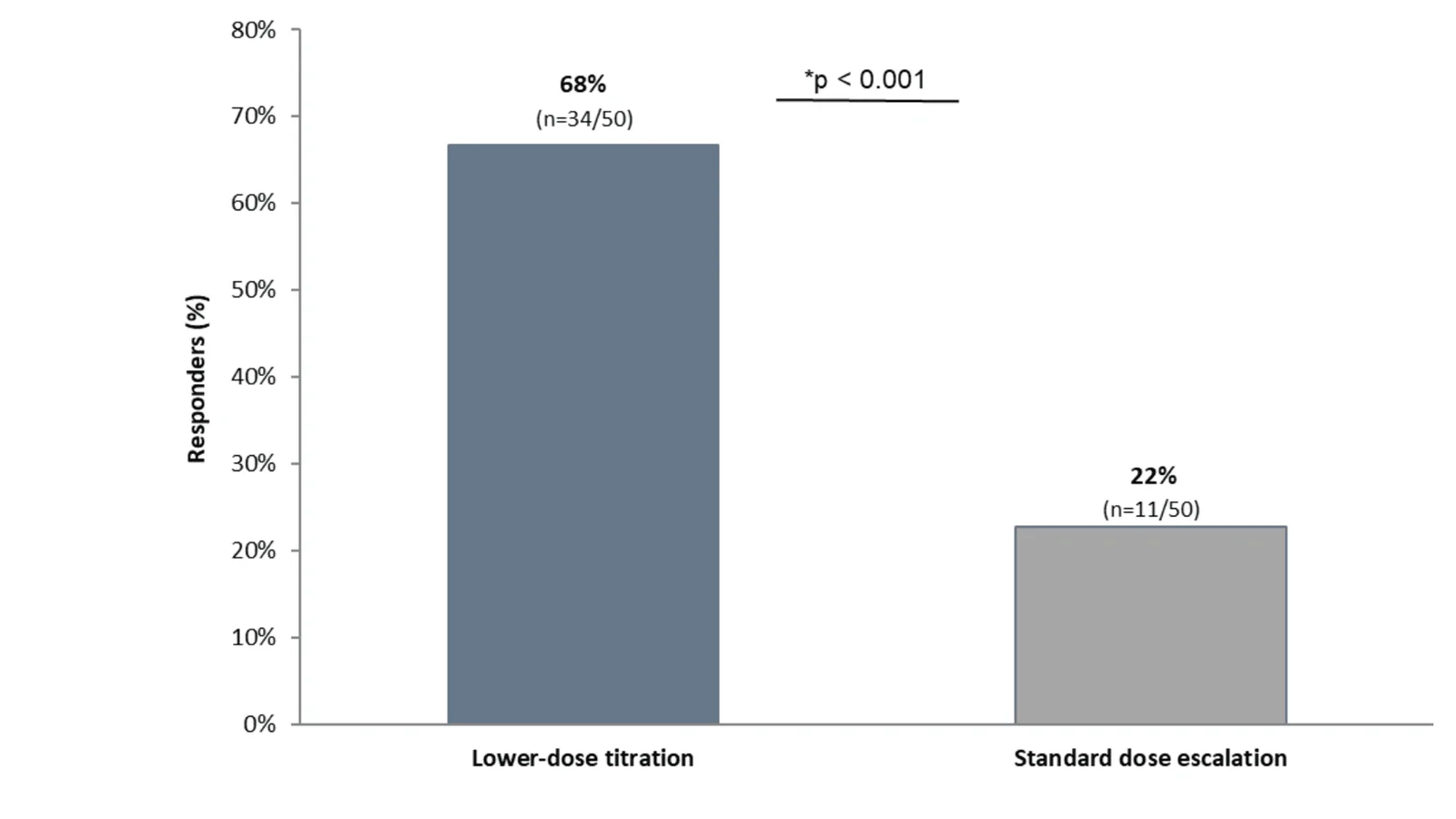
Tirzepatide dosing regimens over the 12-week follow-up period. Each group included 50 patients. Responders were defined as participants achieving a ≥5% reduction in fat mass while maintaining skeletal muscle mass loss below 5% over 12 weeks. Between-group responder proportions were compared using Fisher’s exact test. *Indicates a statistically significant between-group difference (p < 0.001).

Changes in anthropometric measurements

Significant reductions in waist, thigh, and arm circumferences were observed from baseline to 12 weeks in both dosing groups (all p < 0.001). No statistically significant between-group differences were detected in changes in waist (p = 0.727), thigh (p = 0.262), or arm circumference (p = 0.052). These findings should not be interpreted as evidence of equivalence between dosing regimens (Table 3).

**Table 3:** Changes in anthropometric measurements from baseline to 12 weeks according to tirzepatide titration strategy. Data are presented as mean change ± standard deviation (SD). Negative values indicate reductions from baseline to 12 weeks. Analyses were based on available cases for each outcome. Statistical significance was set at p < 0.05.

Outcome	Lower-dose Δ mean ± SD (cm)	Standard-dose Δ mean ± SD (cm)	p-value
Waist circumference	−7.21 ± 3.87	−7.03 ± 3.19	0.727
Thigh circumference	−4.87 ± 2.61	−4.11 ± 1.93	0.262
Arm circumference	−3.23 ± 3.81	−2.39 ± 1.39	0.052

Changes in skinfold thickness

Statistically significant reductions in thigh, abdominal, and triceps skinfold thickness were observed from baseline to 12 weeks in both dosing groups (all p < 0.001). No statistically significant between-group differences were detected in changes in thigh (p = 0.159), abdominal (p = 0.452), or triceps skinfold thickness (p = 0.076). These findings do not establish equivalence between dosing regimens (Table 4).

**Table 4:** Changes in skinfold thickness from baseline to 12 weeks according to tirzepatide titration strategy. Data are presented as mean change ± standard deviation (SD). Negative values indicate reductions from baseline to 12 weeks.

Outcome	Lower-dose Δ mean ± SD (mm)	Standard-dose Δ mean ± SD (mm)	p-value
Thigh skinfold	−7.87 ± 3.49	−6.67 ± 2.23	0.159
Abdominal skinfold	−6.47 ± 5.99	−5.94 ± 1.97	0.452
Triceps skinfold	−6.66 ± 3.07	−5.92 ± 3.00	0.076

## Discussion

In this retrospective observational study, both tirzepatide dosing regimens were associated with reductions in body weight and adiposity over 12 weeks in women with lipedema. No statistically significant between-group differences were detected in changes in body weight or fat mass, although this does not establish equivalence between regimens. The lower-dose group exhibited a smaller reduction in BIA-estimated skeletal muscle mass and a higher proportion of participants meeting the study-defined exploratory composite responder criterion. These observations highlight the importance of evaluating individual body-composition components during pharmacological weight management rather than relying exclusively on total weight reduction.

Previous studies have demonstrated that tirzepatide-associated weight loss involves reductions in both fat mass and lean mass [9-11]. Evidence regarding incretin-based therapies encompasses different clinical contexts, including investigations into the pharmacological rationale for GIP/GLP-1 co-agonism and clinical trials involving tirzepatide in type 1 and type 2 diabetes and obesity [14-16]. The heterogeneity of these populations and approaches limits the direct extrapolation of findings to women with lipedema. The interpretation of weight loss should also consider metabolic adaptations, such as adaptive thermogenesis, and the contribution of skeletal muscle to protein metabolism and metabolic health [17-18]. The clinical benefits of weight reduction may vary according to its magnitude in individuals with obesity. However, this evidence does not establish the clinical significance of the body-composition differences observed between the evaluated regimens [19].

The distinctive characteristics of adipose tissue distribution in lipedema highlight the relevance of investigating regional body-composition changes [5,8]. Although adipose tissue distribution is associated with distinct metabolic implications, the anthropometric and skinfold measurements used in this study do not allow direct quantification of regional fat changes or establish a preferential response to either regimen [20]. Metabolic, inflammatory, and fibrotic mechanisms have also been proposed as potential pathways through which tirzepatide may influence lipedema-related processes [21].

Limitations and future directions

The interpretation of the findings should consider the limitations of the retrospective, non-randomized design, particularly the possibility of confounding by indication, as treatment regimens were selected according to physician judgment and patient tolerability. The lack of adjustment for factors such as physical activity, dietary intake, protein consumption, and lipedema severity means that their potential influence on the observed associations cannot be excluded. Differences in cumulative prescribed tirzepatide exposure also prevent determination of the independent contribution of dose escalation to the observed changes. Additionally, the indirect estimation of skeletal muscle mass using BIA and the absence of objective functional assessments limit the clinical and temporal interpretation of the findings. Nevertheless, the observed associations contribute to characterizing body-composition changes during treatment with different tirzepatide dosing regimens in women with lipedema and provide an exploratory basis for prospective controlled investigations.

## Conclusions

This retrospective observational study found that both tirzepatide dosing regimens were associated with reductions in body weight and fat mass in women with lipedema, with no statistically significant between-group differences detected in the magnitude of these changes. The lower-dose regimen was associated with a smaller reduction in BIA-estimated skeletal muscle mass and a higher observed proportion of participants meeting the study-defined exploratory composite responder criterion. However, the non-randomized design, differences in cumulative drug exposure, and potential residual confounding preclude causal interpretation of these findings. Prospective controlled studies incorporating more direct assessments of body composition and objective functional outcomes are needed to determine whether different tirzepatide dosing regimens are associated with clinically meaningful differences in muscle mass and functional outcomes in women with lipedema.
